# Development of an SA/XLG Composite Hydrogel Film for Customized Facial Mask Applications

**DOI:** 10.3390/polym17172410

**Published:** 2025-09-05

**Authors:** Su-Mei Huang, Xu-Ling Sun, Chia-Ching Li, Jiunn-Jer Hwang

**Affiliations:** 1 Department of Health and Nutrition & Chemical Engineering, Army Academy, Taoyuan 320316, Taiwan; huangsumei888@hotmail.com; 2 Department of Cosmetic Science, Vanung University, Taoyuan 320313, Taiwan; mybaby20051173@gmail.com; 3 Center for General Education, Chung Yuan Christian University, Taoyuan 320314, Taiwan

**Keywords:** sodium alginate, Laponite^®^ XLG, composite hydrogel, biodegradable hydrogel film, personalized fabrication, 3D scanning, skin-contact application

## Abstract

This study aims to address the poor extensibility, brittleness, and limited hydration stability of pure sodium alginate (SA) hydrogels, which hinder their use in flexible, skin-adherent applications such as facial masks, by developing bio-based composites incorporating five representative functional additives: xanthan gum, guar gum, hydroxyethyl cellulose (HEC), poly(ethylene glycol)-240/hexamethylene diisocyanate copolymer bis-decyl tetradeceth-20 ether (GT-700), and Laponite^®^ XLG. Composite hydrogels were prepared by blending 1.5 wt% SA with 0.3 wt% of each additive in aqueous humectant solution, followed by ionic crosslinking using 3% (*w*/*w*) CaCl_2_ solution. Physicochemical characterization included rotational viscometry, uniaxial tensile testing, ATR-FTIR spectroscopy, swelling ratio analysis, and pH measurement. Among them, the SA/XLG composite exhibited the most favorable performance, showing the highest viscosity, shear-thickening behavior, and markedly enhanced extensibility with an elongation at break of 14.8% (compared to 2.5% for neat SA). It also demonstrated a mean swelling ratio of 0.24 g/g and complete dissolution in water within one year. ATR-FTIR confirmed distinct non-covalent interactions between SA and XLG without covalent modification. The hydrogel also demonstrated excellent conformability to complex 3D surfaces, consistent hydration retention under centrifugal stress (+23.6% mass gain), and complete biodegradability in aqueous environments. Although its moderately alkaline pH (8.96) may require buffering for dermatological compatibility, its mechanical resilience and environmental responsiveness support its application as a sustainable, single-use skin-contact material. Notably, the SA/XLG composite hydrogel demonstrated compatibility with personalized fabrication strategies integrating 3D scanning and additive manufacturing, wherein facial topography is digitized and transformed into anatomically matched molds—highlighting its potential for customized cosmetic and biomedical applications.

## 1. Introduction

**Figure 1:**
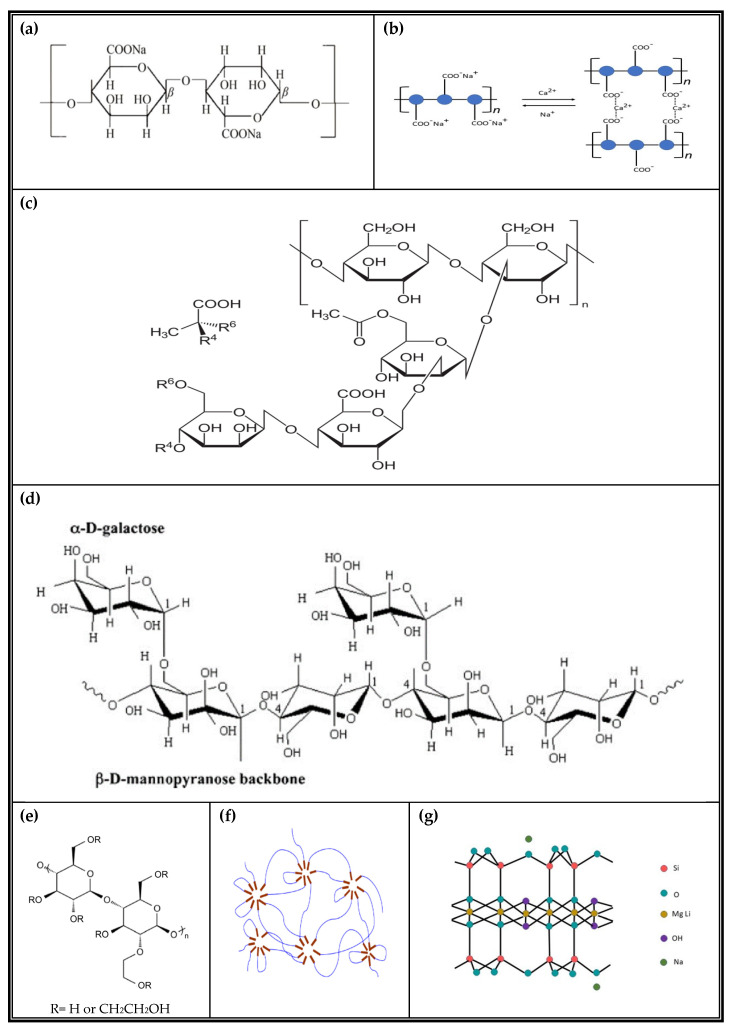
Molecular structures and schematic representations of sodium alginate and related additives: (**a**) sodium alginate (SA) polymer chain; (**b**) Ca^2+^ crosslinking mechanism of sodium alginate illustrating the “egg-box” model; (**c**) xanthan gum (XG) molecular structure; (**d**) guar gum (GG) molecular structure; (**e**) hydroxyethyl cellulose (HEC) repeating unit; (**f**) GT-700 polymer network schematic; (**g**) theoretical crystal structure of Laponite^®^ XLG.

Sodium alginate (SA) is a naturally occurring anionic polysaccharide primarily derived from brown algae and certain bacterial strains (Figure 1a) [1,2]. SA exhibits excellent biocompatibility, biodegradability, and hydrophilicity [3]. These features enable the formation of three-dimensional hydrogel networks via ionic crosslinking with divalent cations—most notably calcium ions (Ca^2+^)—under mild conditions, thereby preserving the biological activity of encapsulated agents (Figure 1b) [3,4]. Owing to these favorable properties, SA-based hydrogels have been extensively explored in diverse fields, including food processing [5,6], biomedical engineering [7,8,9], cosmetics and cosmeceuticals [10,11], bioengineering [12], and environmental engineering [13,14,15,16]. The material’s versatility, safety, and environmental compatibility have contributed to a growing body of research aimed at expanding its practical applications [17].

Despite these advantages, hydrogels composed solely of SA often suffer from limited mechanical strength, low extensibility, and brittleness upon drying. These shortcomings restrict their functionality in applications requiring mechanical resilience, prolonged adhesion, or flexibility under deformation—such as wound dressings, transdermal patches, and facial masks [18]. To overcome these limitations, various strategies have been developed, including composite hydrogel formulation with cationic polysaccharides such as chitosan for electrostatic interactions, proteins like gelatin for hydrogen bonding, or polyvinyl alcohol (PVA) for physical crosslinking via freeze–thaw cycles to enhance tensile strength and elasticity [19,20]; multivalent metal ionic crosslinking using Ca^2+^, Ba^2+^, or Al^3+^ to form stable “egg-box” junctions [21,22]; nanofiller reinforcement with nanoscale mineral fillers such as palygorskite (PAL) and montmorillonite (MMT) to improve structural integrity [23,24]; and double-network (DN) structures combining rigid and ductile networks to increase toughness [25,26]. These strategies enhance tensile strength, elasticity, and toughness, and can be further improved by incorporating plasticizers such as glycerol or sorbitol to reduce cohesive energy between polymer chains, thereby increasing flexibility, extensibility, and tensile strength in SA-based hydrogel films [27,28].

Building upon these strategies, this study incorporates five representative additives—xanthan gum (XG), guar gum (GG), hydroxyethyl cellulose (HEC), GT-700, and Laponite^®^ XLG—selected for their complementary physicochemical properties, such as rheological modulation, film-forming ability, moisture retention, and structural enhancement. A concise comparison of sodium alginate (SA) and its auxiliary additives is illustrated in Figure 1 and summarized in Table 1, which collectively provide a comprehensive overview of their molecular structures or schematic representations (including the Ca^2+^ crosslinking “egg-box” model), sources, key physicochemical properties, and functional roles in enhancing the performance of SA-based hydrogels for facial mask applications. [29,30,31].

The resulting SA-based composite hydrogels were characterized for pH, viscosity, film-forming ability, mechanical strength, and solubility. Potential molecular interactions between SA and each additive were further examined using Fourier transform infrared (FTIR) spectroscopy. Based on the comparative evaluation, the most promising formulation was identified for facial mask applications. Moreover, inspired by the recent literature on biopolymer composites, the optimized composite hydrogel is envisioned to be integrated with three-dimensional (3D) scanning technology in future work as a potential strategy for developing biodegradable and anatomically conformable facial mask carriers [32]. This study aims to develop an SA/XLG composite hydrogel film as a multifunctional, sustainable, and user-adaptive platform for customized facial mask applications. By integrating material innovation with potential 3D scanning technologies, this work seeks to address the evolving requirements of customized facial mask products in the cosmetic field, with potential extension to biomedical applications.

## 2. Experimental Section

### 2.1. Chemicals and Materials

Sodium alginate (Top Rhyme International Co., Ltd., Taipei City, Taiwan); xanthan gum (Top Rhyme International Co., Ltd., Taipei City, Taiwan); guar gum (Shun Yi Chemical Raw Materials Co., Ltd., Taichung City, Taiwan); hydroxyethyl cellulose (HEC) (Top Rhyme International Co., Ltd., Taipei City, Taiwan); PEG-240/hexamethylene diisocyanate copolymer bis-decyl tetradeceth-20 ether (GT-700) (Top Rhyme International Co., Ltd., Taipei City, Taiwan); Laponite^®^ XLG (Top Rhyme International Co., Ltd., Taipei City, Taiwan); citric acid (Chung Yuan Chemicals Inc., Taoyuan City, Taiwan); calcium chloride (Echo Chemical Co., Ltd., Miaoli, Taiwan); ethylenediaminetetraacetic acid tetrasodium salt (EDTA-4Na) (Echo Chemical Co., Ltd., Miaoli, Taiwan); triethanolamine (TEA) (Top Rhyme International Co., Ltd., Taipei City, Taiwan); 1,3-Butylene glycol (Echo Chemical Co., Ltd., Miaoli, Taiwan); glycerin (Shun Yi Chemical Raw Materials Co., Ltd., Taichung City, Taiwan); sodium pyrrolidone carboxylate (NaPCA) (Shun Yi Chemical Raw Materials Co., Ltd., Taichung City, Taiwan); trehalose (Top Rhyme International Co., Ltd., Taipei City, Taiwan); phenoxyethanol (Echo Chemical Co., Ltd., Miaoli, Taiwan).

### 2.2. Instrumentation

High-torque mechanical stirrer (Model: HC-100/B, Her Sheng Chang International Co., Ltd., Taichung City, Taiwan); magnetic stirrer (Model: MG-510, Shibata Scientific Technology Ltd., Soka, Saitama, Japan); electronic balance (Model: ME3002QE, METTLER TOLEDO, Greifensee, Switzerland); RO pure water system (Model: EC-430, Suntex Instruments Co., Ltd., New Taipei City, Taiwan); viscometer (Model: HA-DV3, DV-III Ultra, Brookfield Engineering Laboratories, Middleboro, MA, USA; purchased from Strider Instrument & Application Co., Ltd., Shanghai, China); electronic universal testing machine (Model: EZ-Test, Shimadzu Corporation, Kyoto, Japan; purchased from Sanpany Instruments Co., Ltd., Taipei City, Taiwan); Fourier transform infrared spectrometer (Model: FTS-7, PerkinElmer Inc., Waltham, MA, USA; purchased from PerkinElmer Taiwan Co., Ltd., Taipei City, Taiwan); ultrasonic cleaner (Model: 5510-DTH, Branson Ultrasonics Corporation, Danbury, CT, USA; purchased from Today’s Instruments Co., Ltd., New Taipei City, Taiwan); centrifuge (Model: CN-2160, Hsiangtai Machinery Industry Co., Ltd., New Taipei City, Taiwan); digital pH meter (Model: HI5221-01, HANNA Instruments, Woonsocket, RI, USA; purchased from Biosan Biotech Co., Ltd., Taipei City, Taiwan); drying oven (Model: DH-600N, Yotec Instruments Co., Ltd., New Taipei City, Taiwan); custom-made teak wood molds for casting and drying hydrogel films (fabricated in-house; dimensions: 10.76 × 4.00 × 1.50 cm, L × W × H); custom-made teak wood molds for casting hydrated composite hydrogels (fabricated in-house; dimensions: 3.00 × 3.00 × 0.30 cm and 2.00 × 2.00 × 0.30 cm, L × W × H, respectively); three-dimensional silicone facial mold (unbranded, sourced from a local online retailer).

### 2.3. Preparation of Hydrogels and Crosslinking Solution

#### 2.3.1. Pre-Screening of SA-Based Hydrogels

The compositions of SA-based hydrogels are summarized in Table 2. Each formulation contained sodium alginate at different concentrations (0.5–2.0% *w*/*w*), dissolved in 1,3-butylene glycol and reverse osmosis (RO) water, with phenoxyethanol (0.1 g) as a preservative. All mixtures (final weight 100 g) were stirred at 500 rpm for 30 min at ambient temperature (25 ± 2 °C) until complete dissolution. Based on flowability and film-forming properties, the 1.5% SA solution was selected for subsequent composite hydrogel formulations.

#### 2.3.2. Preparation of SA-Based Composite Hydrogels

Five SA-based composite hydrogel formulations—SA/XG, SA/GG, SA/HEC, SA/GT, and SA/XLG—were prepared according to the compositions summarized in Table 3. Each formulation contained 1.5% sodium alginate and 0.3% of a selected auxiliary agent. In addition, all formulations included 1,3-butylene glycol (6.0 g) as a humectant and phenoxyethanol (0.1 g) as a preservative. For pH adjustment, citric acid (0.1 g) was added to the GG formulation, whereas triethanolamine (0.1 g) was added to the HEC formulation. Reverse osmosis (RO) water was used to adjust the total weight to 100 g. The mixtures were stirred at 500 rpm for 30 min at ambient temperature (25 ± 2 °C) until fully dissolved.

#### 2.3.3. Preparation of Calcium-Based Facial Mist for Ionic Crosslinking

A calcium-based facial mist was prepared as the ionic crosslinker for both sodium alginate hydrogels and SA-based composite formulations, using the composition summarized in Table 4**.** Calcium chloride (3.0 g) was dissolved in a humectant mixture of 1,3-butylene glycol (6.0 g) and glycerin (6.0 g) at ambient temperature (25 ± 2 °C). Subsequently, trehalose (1.0 g) and sodium pyrrolidone carboxylate (NaPCA, 3.0 g) were incorporated to enhance moisture retention. RO water was added to adjust the total weight to 99.9 g. The solution was stirred at 500 rpm for 30 min using a high-torque mechanical stirrer to ensure homogenization. Finally, phenoxyethanol (0.1 g) was added as a preservative, resulting in a final mist weight of 100 g with a calcium chloride concentration of 3% (*w*/*w*).

### 2.4. Flow Behavior Analysis

The flow behavior of the SA-based hydrogel (as described in Section 2.3.1) and five SA-based composite hydrogel formulations—SA/XG, SA/GG, SA/HEC, SA/GT, and SA/XLG (as described in Section 2.3.2)—was evaluated using a rotational viscometer equipped with an SC21 spindle. For each formulation, 7.1 mL of hydrogel was loaded into a cylindrical sample tube and tested at ambient temperature (25 ± 2 °C). Shear stress values were recorded over a range of shear rates by adjusting the spindle rotation speed. All measurements were performed in triplicate, and the resulting flow curves were plotted for comparative evaluation.

### 2.5. Preparation and Characterization of Dried Hydrogel Films

#### 2.5.1. Film Formation and Drying

**Figure 2:**
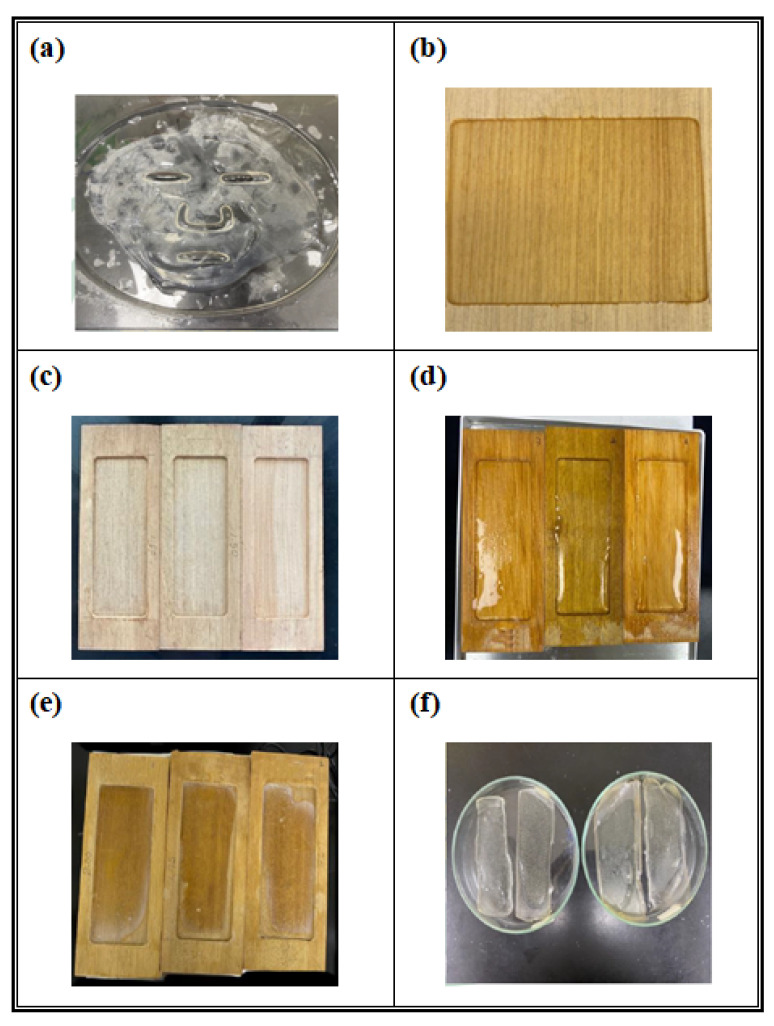
(**a**,**b**) Comparison of hydrogel–mold interactions using different mold materials: (**a**) polymeric hydrogel film showing circumferential shrinkage and contour deformation when cast on a plastic mold; (**b**) uniform spreading and enhanced mold conformity of the hydrogel film on a teak wood mold. (**c**–**f**) Stepwise preparation of SA/XG composite hydrogel films using a custom-fabricated teak wood mold: (**c**) empty wooden mold prior to treatment; (**d**) mold surfaces pre-sprayed with a calcium-based mist, followed by pouring of the SA/XG hydrogel formulation into the cavities and additional surface spraying to initiate ionic crosslinking; (**e**) hydrogel films after 2 h of drying; (**f**) final dried films after 3 h, demolded for subsequent characterization.

Dried hydrogel films of the SA-based hydrogel (Section 2.3.1) and five SA-based composite formulations—SA/XG, SA/GG, SA/HEC, SA/GT, and SA/XLG (Section 2.3.2)—were prepared by casting each formulation into custom-made teak wood molds (10.76 × 4.00 × 1.50 cm, L × W × H) pretreated with a calcium-based mist (Section 2.3.3) at ambient temperature (25 ± 2 °C). After casting, the surface of each formulation was uniformly sprayed with the same calcium mist to promote ionic surface crosslinking. Teak wood molds were selected instead of plastic molds due to their favorable surface hydrophilicity and moisture-absorbing properties, which helped minimize shrinkage and improve film uniformity during casting and drying, as shown in Figure 2a,b.

The filled molds were then placed in a convection oven and dried at 60 °C for 3 h. A visual inspection was conducted after 2 h to assess surface dryness and structural integrity. Representative photographs of the SA/XG formulation were taken at 2 h and 3 h to document the progression of film formation. Upon completion of drying, the films were carefully demolded and stored for subsequent mechanical and spectroscopic analyses. Figure 2c–f presents representative steps of the film preparation process, using the SA/XG formulation as an illustrative example. All formulations were prepared in triplicate to ensure reproducibility.

To simulate practical facial mask application, the SA/XLG formulation (Section 2.3.2) was applied onto a commercially available three-dimensional silicone facial mold (unbranded, sourced from a local online retailer). The outer convex surface of the mold, representing the contours of a human face, was first pretreated with a calcium-based mist (Section 2.3.3). A uniform layer of the composite hydrogel was then spread over the surface, followed by a second spray of the same calcium mist to promote ionic surface crosslinking. No drying step was performed in this procedure. This process was intended to evaluate the hydrogel’s spreadability and conformability on complex three-dimensional surfaces.

#### 2.5.2. Tensile Strength Test

**Figure 3:**
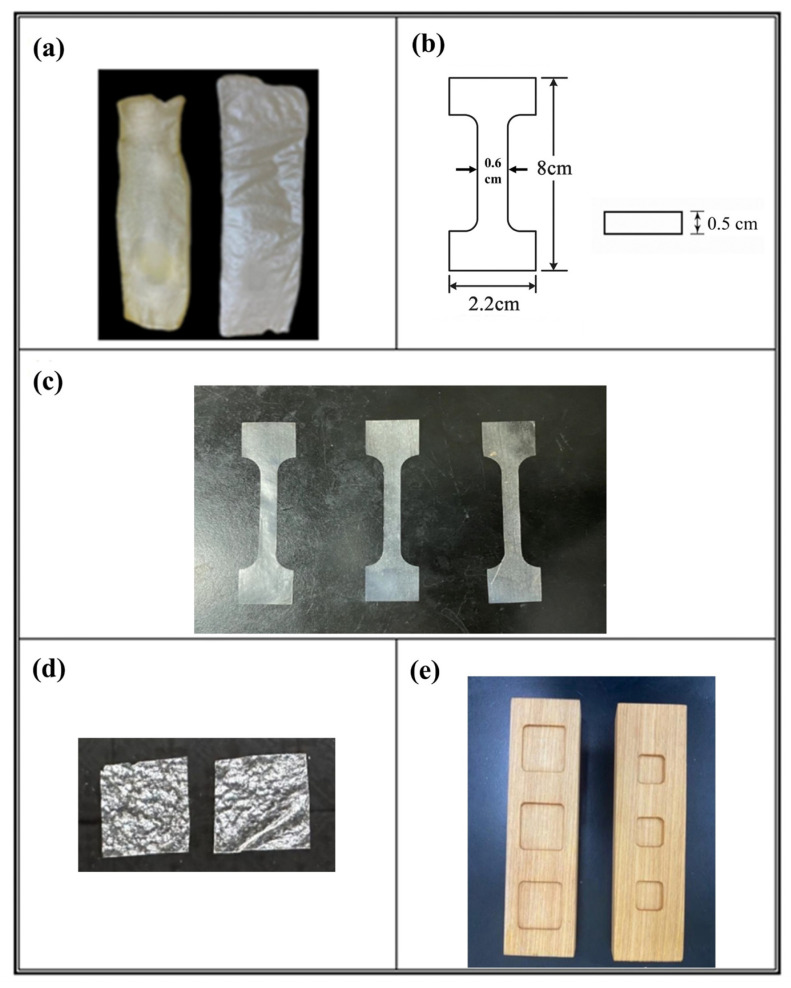
(**a**) Dried hydrogel films: SA film (**left**) and SA/XG composite film (**right**); (**b**) schematic representation of the dumbbell-shaped specimen: front view (8.0 × 2.2 cm, L × W) with a narrowed gauge width of 0.6 cm (**left**) and side view showing a thickness of 0.5 cm (**right**); (**c**) dumbbell-shaped tensile specimens cut from SA/XG composite films; (**d**) representative 2.0 × 2.0 cm SA/XG hydrogel film specimens used for ATR-FTIR analysis; (**e**) custom-made teak molds for hydrogel casting with cavity dimensions of 3.0 × 3.0 × 0.3 cm (**left**) and 2.0 × 2.0 × 0.3 cm (**right**) (L × W × H).

Representative images of the dried SA and SA/XG hydrogel films are shown in Figure 3a to illustrate the visual appearance of the samples following the film formation process. Dried hydrogel films prepared as described in Section 2.5.1 were cut into dumbbell-shaped specimens with dimensions of 8.0 × 2.2 × 0.5 cm (L × W × T) in accordance with ASTM D638 (Figure 3b). Figure 3c shows a representative image of the actual SA/XG specimens after cutting. This standardized geometry was employed to ensure consistency across all tensile tests. Tensile tests were performed using a universal testing machine equipped with a load cell suitable for soft polymeric materials, operating at a constant crosshead speed under ambient laboratory conditions (25 ± 2 °C). Force–time curves were recorded for each measurement, from which the maximum tensile strength and extension at break were determined to assess the mechanical performance of each sample.

#### 2.5.3. Method of ATR-FTIR Spectral Analysis

Square specimens (2.0 × 2.0 cm), cut from dried hydrogel films as described in Section 2.5.1 and shown in Figure 3d, were analyzed using a Fourier transform infrared (FTIR) spectrometer equipped with a diamond attenuated total reflection (ATR) accessory. Spectra were acquired in the range of 4000–500 cm^−1^ at a resolution of 4 cm^−1^, with 128 scans averaged per sample. The obtained spectra were used to identify characteristic absorption bands and to assess potential molecular interactions or compositional variations within the sodium alginate-based hydrogel formulations.

#### 2.5.4. Application of SA/XLG Hydrogel on 3D Facial Mold

Additionally, to evaluate the practical applicability of the SA/XLG hydrogel formulation (Section 2.3.2), a wet application test was performed using a commercially available three-dimensional silicone facial mold (unbranded, sourced from a local online retailer). The outer convex surface of the mold, representing the contours of a human face, was first pretreated with a calcium-based mist (Section 2.3.3). A uniform layer of the SA/XLG hydrogel was then spread over the surface, followed by a second spray of the same calcium mist to promote ionic surface crosslinking. No drying step was performed in this procedure. This process was intended to evaluate the hydrogel’s spreadability and conformability on complex three-dimensional surfaces. Subsequent physicochemical analyses of the SA/XLG hydrogel films were conducted to further characterize their aqueous solubility, swelling behavior, and pH suitability for skin application, as described in Section 2.6.

### 2.6. Physicochemical Characterization of the SA/XLG Composite Hydrogel Film

The SA/XLG formulation was subjected to further physicochemical characterization based on the protocols described in Section 2.4 and Section 2.5.2. To investigate its environmental behavior, the solubility and swelling properties of the dried film were evaluated under aqueous conditions. Additionally, the pH of the hydrogel formulation was measured to assess its potential dermatological compatibility. These analyses were performed to characterize the hydrogel’s aqueous behavior and pH profile, both of which are critical parameters for its potential application as a single-use, water-responsive material for topical or skin-contact use.

#### 2.6.1. Solubility and Swelling Behavior

Two sizes of wet SA/XLG hydrogel films were prepared for solubility and swelling tests based on the general procedure described in Section 2.5.1, with modifications to the casting volume and mold dimensions. All casting and pretreatment steps were carried out at ambient temperature (25 ± 2 °C) prior to subsequent stirring, ultrasonic treatment, or centrifugation. Prior to casting, the teak wood molds were pretreated with a calcium-based mist (Section 2.3.3) to facilitate ionic crosslinking. Film (a) was cast by dispensing 2.0 mL of the SA/XLG formulation (Section 2.3.2) into molds measuring 3.0 × 3.0 × 0.3 cm (L × W × H), as shown in Figure 3e (left), whereas film (b) was prepared by casting 1.0 g of the formulation into smaller molds measuring 2.0 × 2.0 × 0.3 cm, as shown in Figure 3e (right). The surface of each film was subsequently sprayed with the same calcium-based mist to promote additional surface crosslinking. All films were tested in their wet, uncured state.

To simulate post-use aqueous environments, three agitation conditions were applied using different film types: (1) film (a) was immersed in 100 mL of reverse osmosis (RO) water and stirred using a magnetic stirrer at 50 rpm for 3 h; (2) film (a) was immersed in 100 mL of RO water and agitated with a high-torque mechanical stirrer at 500 rpm for 3 h; and (3) film (b) was immersed in RO water at a 1:10 (*w*/*v*) ratio, treated using an ultrasonic cleaner for 3 h, and then centrifuged at 3000 rpm for 30 min. After treatment, samples were left to stand at ambient temperature for 24 h. Samples from conditions (1) and (2) were filtered and visually inspected, while those from condition (3) were examined directly in the centrifuge tube.

To assess water absorption and long-term dispersibility, additional film (b) samples were placed into empty centrifuge tubes and weighed to obtain their initial mass. RO water was added to fully immerse the samples, followed by agitation in a centrifuge mixer for 3 h. After filtration to remove excess water, the tubes containing the swollen samples were reweighed to determine mass gain. The samples were then transferred to a sealed glass container containing 1000 mL of RO water and stored at ambient temperature for one year. After storage, physical integrity was evaluated by visual inspection.

#### 2.6.2. pH Measurement

The pH of freshly prepared SA/XLG hydrogel samples was measured using a calibrated digital pH meter at ambient temperature (25 ± 2 °C). Measurements were conducted in triplicate, and the mean value was recorded.

## 3. Results and Discussion

### 3.1. Physicochemical Properties of SA-Based Composite Hydrogels

#### 3.1.1. Flow Behavior and Viscosity Analysis

**Figure 4:**
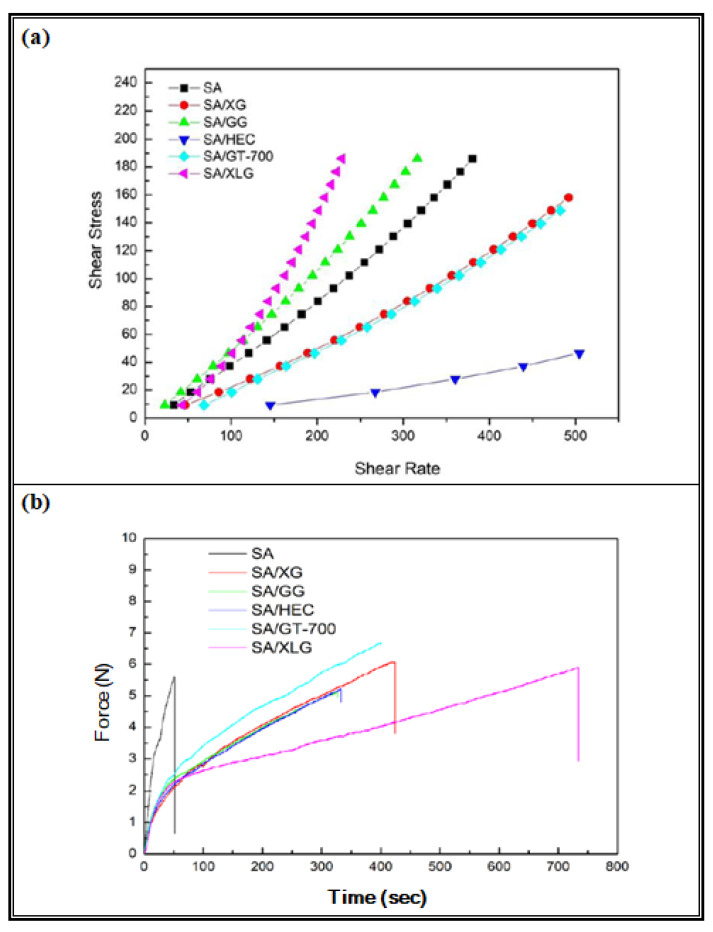
(**a**) Shear stress–shear rate profiles and (**b**) tensile force–time curves of sodium alginate (SA) and SA-based composite hydrogel formulations, including SA/XG, SA/GG, SA/HEC, SA/GT-700, and SA/XLG.

As described in Section 2.4, the flow behavior of the SA-based hydrogel and five SA-based composite formulations was evaluated using a rotational viscometer. As shown in Figure 4a, all samples exhibited non-Newtonian flow behavior with distinct shear-dependent responses. Among them, SA/XLG exhibited the highest shear stress and a characteristic shear-thickening response, indicating enhanced internal resistance and a more cohesive gel network. In contrast, SA/HEC showed the lowest viscosity and typical shear-thinning behavior, suggesting limited network formation. The viscosity ranking was SA/XLG > SA/GG > SA > SA/XG > SA/GT-700 > SA/HEC. While the incorporation of guar gum (GG) also improved viscosity to a certain extent, the effect was more pronounced with Laponite^®^ XLG, which exhibited the highest rheological strength and structural coherence among the tested formulations. These rheological properties, particularly in the SA/XLG formulation, are expected to support improved mechanical strength and facilitate uniform film formation, as further examined in the subsequent sections.

#### 3.1.2. Mechanical Strength and Extensibility

To further assess structural integrity, the maximum extension, elongation at break, and tensile strength of dried sodium alginate (SA)-based hydrogel films were evaluated using uniaxial tensile testing. The resulting tensile force–time curves and corresponding maximum extension and elongation at break values are presented in Figure 4b and Table 5, respectively. The pure SA film exhibited the lowest extension (2.02 mm), the lowest elongation at break (2.53%), and the lowest tensile strength (0.187 MPa), and fractured rapidly upon loading, indicating poor ductility and insufficient structural cohesion.

In contrast, all composite formulations demonstrated markedly improved tensile properties. SA/GT-700 exhibited the highest tensile force, reflecting an enhanced capacity to withstand mechanical loads prior to failure. SA/XLG showed the greatest elongation at break (14.80%), followed by SA/XG (10.38%), SA/HEC (9.20%), SA/GT-700 (8.99%), and SA/GG (7.60%). SA/XLG also exhibited a moderate yet notable tensile strength (0.193 MPa), indicating a favorable balance between strength and extensibility. SA/XG displayed relatively high extensibility but moderate tensile strength. SA/HEC showed balanced performance, with intermediate values in both parameters. In contrast, SA/GG had the lowest extensibility and a tensile strength similar to SA/HEC, indicating comparable strength but reduced flexibility.

Considering the elastic response, the elastic modulus (E) (see Table 5) was determined from the initial linear segment (≤0.5% strain) of the stress–strain curves. The values were: SA 20.67 MPa, SA/XG 13.31 MPa, SA/GT-700 11.47 MPa, SA/GG 11.34 MPa, SA/XLG 11.14 MPa, and SA/HEC 6.91 MPa. These findings indicate that neat SA exhibits the highest stiffness, whereas composite films reduce stiffness to varying degrees while enhancing extensibility, yielding a more compliant and skin-friendly behavior. The stiffness ranking was broadly SA > SA/XG ≳ SA/GT-700 ≈ SA/GG ≈ SA/XLG > SA/HEC, which reflects the intrinsic trade-off between stiffness and elongation at break.

Overall, these results indicate that the incorporation of different additives modulates the tensile behavior of SA-based hydrogel films to varying extents. Comparisons with the literature further reveal that dried alginate-based films can achieve substantially higher strength under certain formulations. For example, dried calcium–alginate films reinforced with soy protein isolate or pectin at 57% RH exhibited tensile strengths of 24.5–38.1 MPa with limited elongation (3.9–5.3%) [33]; these data were obtained from Table 5 of Harper’s thesis. In contrast, nanocellulose–alginate composite dry films have been reported to reach tensile strengths of approximately 44 MPa with an elongation at break of 16.3% in the dry state [34]. Within this context, the SA/XLG dried films developed in the present study (~0.19 MPa, 14.80% elongation) represent a distinct formulation optimized for flexibility and conformability rather than high load-bearing capacity. Notably, the combined tensile strength and extensibility of SA/XLG underscore its potential suitability for skin-adherent applications such as cosmetic facial masks, where both mechanical durability and wearing comfort are essential.

#### 3.1.3. ATR-FTIR Spectral Analysis

**Figure 5:**
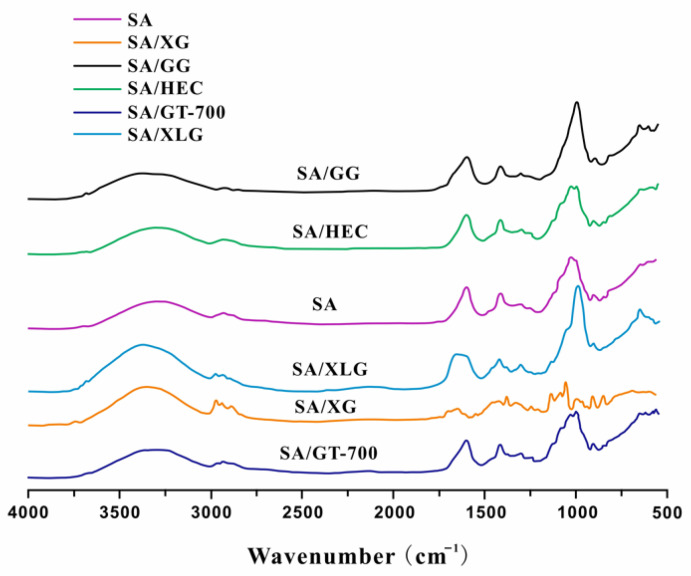
ATR-FTIR spectra of SA and SA-based composite hydrogel films (SA/XG, SA/GG, SA/HEC, SA/GT-700, and SA/XLG).

As described in Section 2.5.3, ATR-FTIR spectroscopy was performed to examine potential molecular interactions between sodium alginate (SA) and the incorporated additives. Figure 5 presents the overlaid ATR-FTIR spectra of the SA-based film and composite formulations incorporating xanthan gum (SA/XG), guar gum (SA/GG), hydroxyethyl cellulose (SA/HEC), GT-700 (SA/GT-700), and Laponite^®^ XLG (SA/XLG). All samples displayed a broad absorption band centered at approximately 3300 cm^−1^, corresponding to O–H stretching vibrations, along with two well-defined peaks near 1600 and 1410 cm^−1^, attributable to the asymmetric and symmetric stretching of carboxylate (–COO^−^) groups, respectively. Additional absorption features in the region of 1000–500 cm^−1^ were assigned to C–O–C stretching and skeletal vibrations of the polysaccharide backbone.

Relative to the pure SA film, the composite samples exhibited minor wavenumber shifts and subtle but observable differences in peak intensity and sharpness, particularly in the hydroxyl (∼3300 cm^−1^) and carboxylate (∼1600 and 1410 cm^−1^) regions. These spectral changes are indicative of non-covalent interactions between SA and the respective additives, without any evidence of covalent bond formation. This suggests that no side reactions occurred during the film formation process, thereby preserving the molecular integrity of the original components. Among all formulations, the SA/XLG film demonstrated the most distinct spectral modifications. The O–H stretching band appeared sharper and slightly redshifted, accompanied by an enhanced absorption near 1040 cm^−1^. These spectral features are consistent with more pronounced intermolecular interactions in the SA/XLG system, a phenomenon also reported in previous studies on layered silicate–polysaccharide composites [35,36]. This interpretation is further corroborated by the elevated viscosity and improved mechanical properties observed in the SA/XLG formulation, as discussed in Section 3.1.1 and Section 3.1.2. Taken together, these spectral observations suggest that the SA/XLG formulation exhibited more pronounced molecular-level interactions than the other tested composites, while maintaining the chemical integrity of its constituents. This supports its potential suitability for applications involving direct skin contact, such as cosmetic facial masks.

### 3.2. Aqueous Behavior and pH Characteristics of SA/XLG Composite Hydrogel

Based on the findings summarized in Section 3.1, the SA/XLG formulation was selected for subsequent evaluation of its aqueous behavior and pH characteristics, as indicators of environmental responsiveness and potential skin compatibility. Rheological and mechanical characterizations (Section 3.1.1 and Section 3.1.2) demonstrated enhanced viscoelasticity, superior extensibility, and improved structural coherence. In addition, ATR-FTIR analysis (Section 3.1.3) revealed distinct molecular-level interactions between sodium alginate and Laponite^®^ XLG, indicative of a physically crosslinked network. The absence of covalent bond formation suggests that the hydrogel matrix is stabilized predominantly through electrostatic interactions and hydrogen bonding, which reduces the risk of undesired chemical by-products and enhances biocompatibility. Taken together, these results support the selection of SA/XLG as a promising candidate for further evaluation of swelling, solubility, and pH profile in the context of sustainable, single-use cosmetic applications.

#### 3.2.1. Swelling and Solubility Behavior

**Figure 6:**
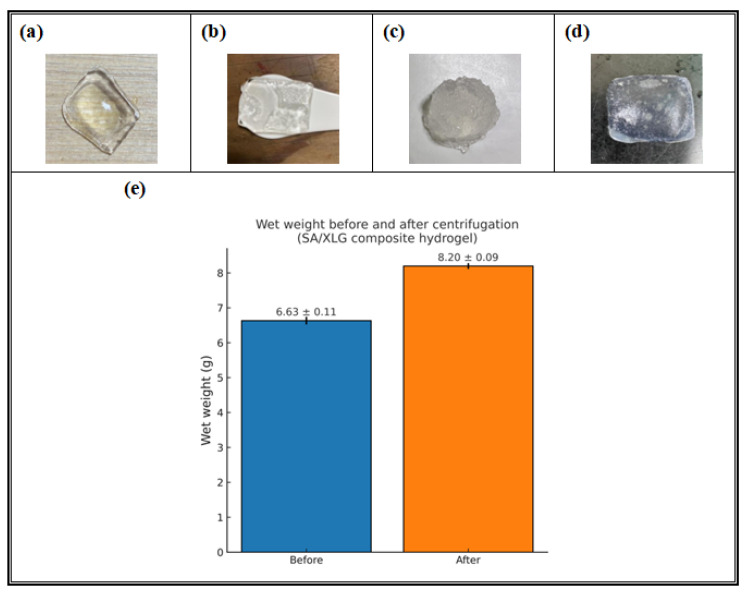
Aqueous stability and swelling behavior of the SA/XLG composite hydrogel. (**a**–**c**) Appearance before and after different agitation conditions: (**a**) untreated; (**b**) after magnetic stirring followed by 24 h of standing and filtration with filter paper; (**c**) after high-torque agitation followed by 24 h of standing and filtration with filter paper. (**d**) Representative image of the untreated SA/XLG hydrogel film (2.0 × 2.0 cm) prior to ultrasonic and centrifugal treatment. (**e**) Wet weight of hydrogel films before and after centrifugation (3000 rpm, 3 h), presented as mean ± SD (before: 6.63 ± 0.11 g; after: 8.20 ± 0.09 g, *n* = 3); error bars indicate the standard deviation from three independent measurements.

According to Section 2.6.1, the swelling and solubility behavior of SA/XLG composite hydrogel films were evaluated under various aqueous conditions to simulate post-use scenarios. As shown in Figure 6a, the untreated hydrogel appeared transparent and structurally intact. After magnetic stirring at 50 rpm for 3 h, followed by 24 h of standing and subsequent filtration (Figure 6b), the hydrogel largely retained its original morphology, with only slight peripheral swelling observed. These findings suggest that the SA/XLG composite hydrogel exhibited good structural stability under mild agitation.

In contrast, high-torque stirring at 500 rpm for 3 h, followed by 24 h of standing and subsequent filtration (Figure 6c), induced pronounced morphological changes. The originally cohesive film structure was disrupted, exhibiting significant swelling and surface fragmentation without visible detachment. These results indicate that the SA/XLG composite hydrogel undergoes partial dispersion when subjected to elevated shear stress, while still maintaining partial structural cohesion. This shear-responsive behavior is indicative of the physically crosslinked nature of the hydrogel network and may be advantageous in contexts where mechanical pretreatment or stress-induced structural breakdown is relevant, such as the handling of single-use biomedical materials or the development of environmentally conscious waste processing strategies.

Ultrasonic-assisted treatment followed by centrifugation provided further insights into the structural resilience of the SA/XLG composite hydrogel film. As shown in Figure 6d, the untreated SA/XLG composite hydrogel film measured 2.0 × 2.0 cm. It was then subjected to continuous ultrasonic exposure for 3 h, followed by centrifugation at 3000 rpm for 30 min. Despite prolonged treatment, the hydrogel appeared visually unchanged. The intact gel film settled at the bottom of the tube, maintaining its integrity and translucency. These observations indicate that although water uptake occurred, structural breakdown was minimal under these combined conditions.

Quantitative evaluation of swelling behavior is shown in Figure 6e. The SA/XLG composite hydrogel films exhibited a consistent increase in wet mass across three replicates following centrifugation. Initial weights ranged from 6.56 to 6.76 g, increasing to 8.10–8.26 g after treatment, corresponding to an average water uptake of 1.57 g. This corresponds to a mean swelling ratio of 0.24 g/g and a relative weight increase of approximately 23.6%. No visible structural degradation or gel detachment was observed. The reproducible swelling behavior under centrifugal stress suggests the presence of an interconnected hydrated network, consistent with previous reports that Laponite–alginate composites form polymer–clay assemblies stabilized by edge–face interactions. Overall, these findings demonstrate the hydrogel’s reproducible swelling behavior and structural stability under prolonged centrifugal stress (3000 rpm for 3 h).

Following the quantitative swelling experiment, the swollen SA/XLG hydrogel was subjected to long-term immersion in RO water under ambient conditions (25 ± 2 °C). Initially, the hydrogel formed a translucent sediment that gradually dispersed during storage. After one year, the sample had completely dissolved, leaving no visible residue in the solution. This observation confirms the hydrogel’s gradual disintegration in aqueous environments and indicates its potential biodegradability, thereby supporting its suitability for environmentally responsive single-use systems. Such long-term dissolution further supports the interpretation of a hydrated polymer–clay network that promotes water transport and ultimately biodegradability.

Collectively, these findings demonstrate that the SA/XLG composite hydrogel exhibits controlled aqueous responsiveness, mechanical robustness, and long-term degradability, making it a promising candidate for environmentally responsible, single-use applications. However, the degradation rate as a function of time was not quantified in this study and should be addressed in future investigations using systematic gravimetric measurements over time, complemented by spectroscopic techniques to elucidate potential structural changes during degradation. In addition, hydration performance in this study was primarily inferred from swelling and solubility behavior, without direct TEWL analysis. Future work will therefore incorporate water retention curves and controlled evaporation experiments, and may also include benchmarking against commercial sheet masks to provide a more rigorous quantitative assessment of hydration behavior.

#### 3.2.2. pH Evaluation and Dermatological Implications

**Figure 7:**
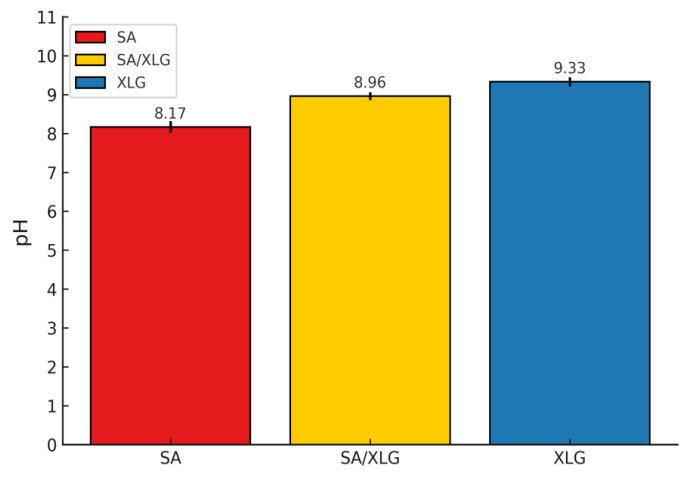
pH values of the SA, XLG, and SA/XLG hydrogel formulations.

As described in Section 2.6.2, the pH values of SA, XLG, and the SA/XLG composite hydrogel were measured to evaluate potential skin compatibility. As shown in Figure 7, the SA/XLG formulation exhibited a pH of 8.96, which was slightly lower than that of XLG alone (9.33), yet still above the optimal physiological skin range (pH 4.5–6.5). A pH in this moderately alkaline range may pose a risk of skin irritation, particularly with prolonged or repeated topical exposure. Therefore, future formulation strategies should consider buffering the pH using weak organic acids (e.g., lactic acid) or naturally derived amino acids to improve dermatological compatibility and expand the formulation’s suitability for cosmetic or skin-contact applications.

### 3.3. Functional Performance and Application-Oriented Adaptability of SA/XLG Composite Hydrogel

#### 3.3.1. Hydrogel Film Formation and Anatomical Mold Adaptability

To assess the moldability of the SA/XLG composite hydrogel for facial mask formation, the complete preparation and molding procedure—including hydrogel application and calcium-induced ionic crosslinking—was carried out as described in the final paragraph of Section 2.5.1. No drying was performed, allowing the hydrogel to retain its hydrated state for conformation assessment.

**Figure 8:**
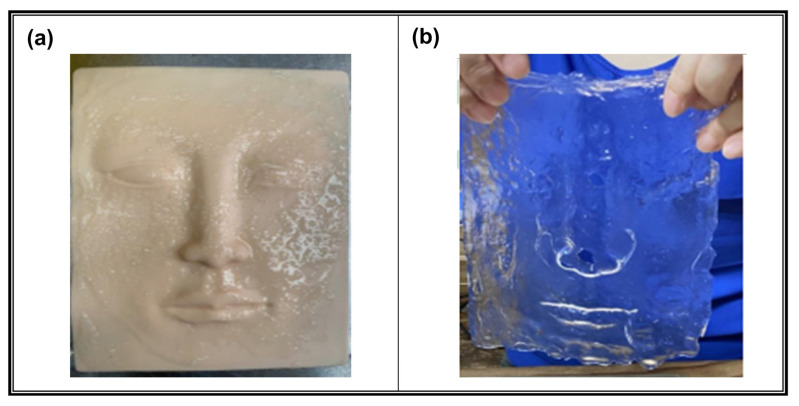
Formation and mold adaptability of the SA/XLG composite hydrogel. (**a**) SA/XLG composite hydrogel conformally crosslinked on the convex surface of a three-dimensional silicone facial mold; (**b**) freestanding SA/XLG hydrogel mask after demolding, retaining full-face.

As shown in Figure 8a, the SA/XLG composite hydrogel conformed closely to the convex surface of the three-dimensional silicone facial mold, replicating detailed anatomical features such as the nose bridge, eye sockets, lips, and overall facial contours. The calcium-induced ionic crosslinking provided sufficient cohesion while preserving the hydrogel’s flexibility in its hydrated state. Upon gentle demolding, the resulting hydrogel mask retained an intact full-face geometry with clear reproduction of surface topography, including fine structural details (Figure 8b).

The freestanding film demonstrated adequate mechanical integrity to be lifted and handled without tearing or deformation, indicating its suitability as a structurally stable, form-fitting facial mask. These results confirm that the SA/XLG composite hydrogel offers a balanced combination of conformability, cohesiveness, and flexibility required for directly cast, application-specific hydrogel masks. Furthermore, the demonstrated mold adaptability underscores its potential for use in customized skin-contact products, where anatomical fit and material integrity are essential.

#### 3.3.2. Prospective Integration with 3D Printing Technologies

Conventional sheet masks are typically produced using fixed-size templates that do not accommodate the anatomical variability of individual users. This often results in overextension, insufficient coverage, or poor adherence over complex facial regions, ultimately compromising user comfort, mask retention, and the effectiveness of topical delivery. To address these limitations, the integration of three-dimensional (3D) scanning and additive manufacturing technologies offers a promising strategy for the development of personalized hydrogel facial masks. Although not experimentally implemented in the present study, the envisioned workflow involves capturing individualized facial geometries via high-resolution scanning, followed by digital modeling and 3D printing of anatomically contoured molds. These customized convex molds could serve as form-fitting templates for fabricating hydrogel films with enhanced facial conformity. In this study, commercially available silicone molds were used to simulate the mold-based production approach, as described in Section 3.3.1.

To support sustainable implementation, future adaptations may incorporate biodegradable and hydrophilic materials for 3D printing, as the importance of mold hydrophilicity was evident in hydrogel behavior observed in this study (Section 2.5.1, first paragraph). Polylactic acid (PLA), a thermoplastic derived from renewable resources, is widely used in FDM-based biomedical and cosmetic applications, including the fabrication of facial devices that demonstrate reliable printability, biocompatibility, and dimensional stability for direct skin-contact use [37,38]. While inherently hydrophobic, its surface hydrophilicity can be enhanced through chemical modification or polymer blending [39,40]. Given these attributes, PLA is well-suited for fabricating 3D molds used in hydrogel facial mask production, while simultaneously contributing to low-waste systems aligned with green manufacturing principles.

Recent studies have demonstrated the feasibility of applying 3D-printed hydrogel systems in personalized skin-contact applications [41,42], supporting the development of versatile hydrogel formulations such as the SA/XLG composite hydrogel evaluated here. This biopolymer-based hydrogel showed reproducible conformability on complex surfaces, laying a foundation for integration with digital manufacturing in personalized skincare.

Building on these findings, the SA/XLG system may also serve broader purposes, including anatomically contoured wound dressings, postoperative facial pads, or transdermal patches. Its water-responsive nature and ease of fabrication render it suitable for single-use applications emphasizing sustainability and custom-fit biomedical care. Future studies will integrate high-resolution 3D facial scanning with digital mold design and additive manufacturing to systematically evaluate how hydrogel viscosity, curing time, and mechanical properties influence the precision and efficiency of personalized mask fabrication.

## 4. Conclusions

This study developed bio-based composite hydrogels to overcome the brittleness and limited flexibility of pure sodium alginate (SA). Among all tested formulations, the SA/XLG composite exhibited the most favorable balance of viscosity, extensibility, and mechanical robustness. ATR-FTIR analysis confirmed non-covalent molecular interactions without chemical modification, supporting its structural stability and biocompatibility. In addition, the SA/XLG hydrogel demonstrated reproducible swelling capacity, strong water retention, and gradual solubility under aqueous conditions, highlighting its potential as a sustainable material for next-generation facial mask applications. Importantly, this work also introduced a conceptual framework integrating 3D facial scanning with additive manufacturing (e.g., PLA-based molds), providing a pathway for the fabrication of anatomically contoured hydrogel masks with improved fit, comfort, and ecological sustainability.

Further studies should systematically quantify degradation kinetics using gravimetric and spectroscopic analyses, benchmark hydration retention against commercial standards, and optimize pH for dermatological compatibility. In addition, comprehensive in-vitro and in vivo biocompatibility evaluations are required, as parameters such as pH, ionic stability, and the absence of chemical side products may critically affect safety. The porosity of the SA/XLG hydrogel, not characterized here, represents another determinant of water transport and active ingredient release and will be investigated using SEM and pore size distribution analysis. Validation under practical skin-wearing conditions and comparison with commercial sheet masks will also be essential for real-world translation. Finally, scaling up eco-friendly production and integrating digital manufacturing workflows with 3D scanning may enable the development of personalized hydrogel structures, extending the applicability of the SA/XLG system to advanced biomedical applications such as wound dressings, postoperative facial pads, and transdermal delivery systems.

## Figures and Tables

**Table 1:** Sources, key physicochemical properties, and functional roles of sodium alginate (SA) and auxiliary additives used in the composite hydrogel formulations.

Additive	Source	Key Physicochemical Properties	Role in This Study
1. Sodium Alginate (SA)	Brown algae/bacteria	Biocompatible, hydrophilic, ionic gelation	Base polymer for hydrogel matrix
2. Xanthan Gum (XG)	Fermentation of Xanthomonas campestris	High viscosity, shear-thinning, stability	Enhances viscosity and membrane uniformity
3. Guar Gum (GG)	Seeds of Cyamopsis tetragonoloba	High hydration capacity, viscoelastic	Improves softness, elasticity, and moisture retention
4. Hydroxyethyl Cellulose (HEC)	Cellulose derivative	Water-soluble, film-forming, stable	Enhances film formation and flexibility
5. GT-700	PEG-240/HDI copolymer surfactant	Nonionic surfactant, improves spreadability	Improves emulsion stability and handling
6. Laponite^®^ XLG	Synthetic layered silicate	Thixotropic, shear-thinning, colloidal stability	Reinforces structure; increases viscosity and extensibility

**Table 2:** Formulations of SA-based hydrogels.

SA Concentration (*w*/*w*, %)	Sodium Alginate (g)	1,3-Butylene Glycol (g)	Phenoxyethanol (g)	RO Water (g)	Total (g)
0.5	0.5	6.0	0.1	93.4	100
1.0	1.0	6.0	0.1	92.9	100
1.5	1.5	6.0	0.1	92.4	100
2.0	2.0	6.0	0.1	91.9	100

**Table 3:** Compositions of the SA-based composite hydrogels.

Formulation	Sodium Alginate (g)	Auxiliary Agent (g)	1,3- Butylene Glycol (g)	pH Regulator (g)	Phenoxyethanol (g)	RO Water (g)
SA/XG	1.5	Xanthan gum (0.3)	6.0	–	0.1	92.1
SA/GG	1.5	Guar gum (0.3)	6.0	Citric acid (0.1)	0.1	92.0
SA/HEC	1.5	Hydroxyethyl cellulose (0.3)	6.0	Triethanolamine (0.1)	0.1	92.0
SA/GT	1.5	GT-700 (0.3)	6.0	–	0.1	92.1
SA/XLG	1.5	Laponite^®^ XLG (0.3)	6.0	–	0.1	92.1

**Table 4:** Composition of calcium-based facial mist (crosslinking solution).

Component	Function	Weight (g)
Calcium chloride	Ionic crosslinker	3.0
1,3-Butylene glycol	Humectant	6.0
Glycerin	Humectant	6.0
Trehalose	Moisture retention	1.0
Sodium pyrrolidone carboxylate (NaPCA)	Moisture retention	3.0
Reverse osmosis (RO) water	Solvent	80.9
Phenoxyethanol	Preservative	0.1
Total	—	100

**Table 5:** Mechanical properties of sodium alginate (SA) and SA-based composite hydrogel films.

Film Formulation	Maximum Extension (mm)	Elongation at Break (%)	Tensile Strength (MPa)	Elastic Modulus, E (MPa)
SA	2.02	2.53	0.187	20.67
SA/XG	8.30	10.38	0.200	13.31
SA/GG	6.08	7.60	0.170	11.34
SA/HEC	7.36	9.20	0.173	6.91
SA/GT-700	7.19	8.99	0.220	11.47
SA/XLG	11.84	14.80	0.193	11.14

Note: Testing performed on dried films at ambient laboratory temperature (25 ± 2 °C).

## Data Availability

Data are contained within the article. Further inquiries can be directed to the corresponding author.
